# Supplementary figures and images for: Effects of low versus high inspired oxygen fraction on myocardial injury after transcatheter aortic valve implantation: A randomized clinical trial
Source: PLoS One. 2023 Aug 2;18(8):e0281232. doi: 10.1371/journal.pone.0281232 (PMC10395822; doi:10.1371/journal.pone.0281232)

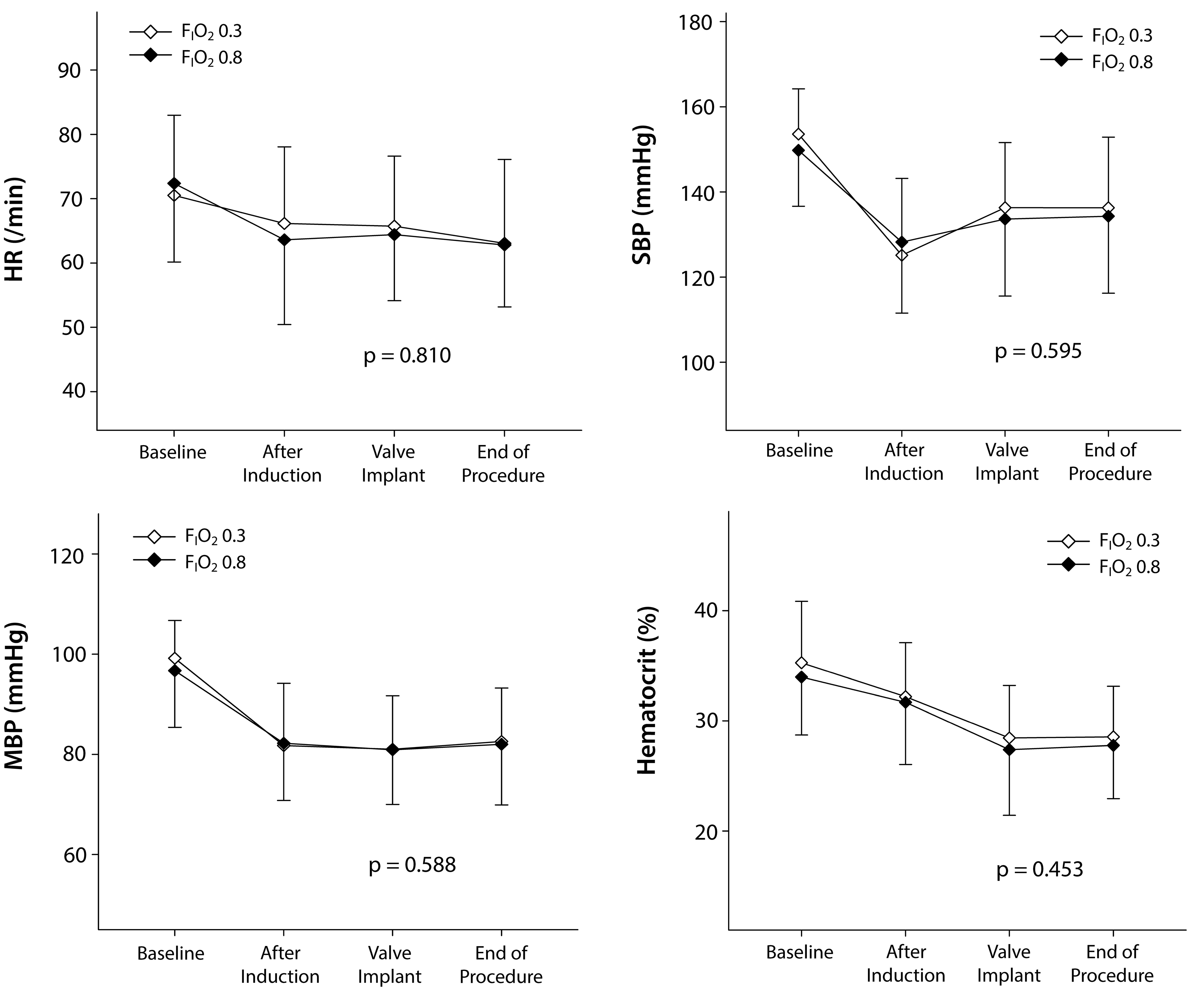

Supplement: S1 Fig — HR, heart rate; MBP, mean blood pressure; SBP, systolic blood pressure. (TIF) [file pone.0281232.s002.tif]

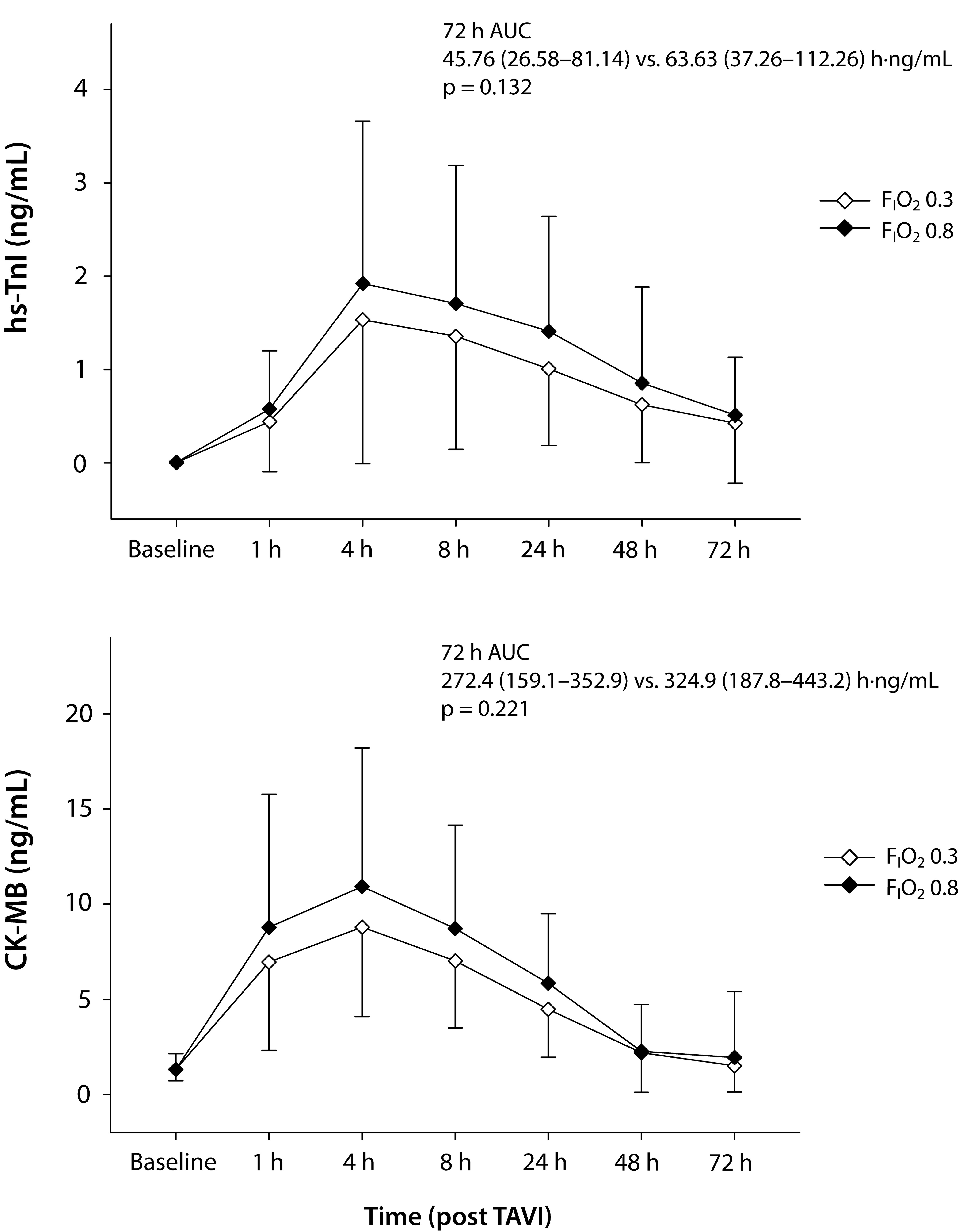

Supplement: S2 Fig — AUC, area under the curve; CK-MB, creatine kinase-myocardial band; hs-cTnI, high sensitivity cardiac troponin I; TAVI, transcatheter aortic valve implantation. (TIF) [file pone.0281232.s003.tif]
