# Supplementary material for: Effects of low versus high inspired oxygen fraction on myocardial injury after transcatheter aortic valve implantation: A randomized clinical trial
Source: PLoS One. 2023 Aug 2;18(8):e0281232. doi: 10.1371/journal.pone.0281232 (PMC10395822; doi:10.1371/journal.pone.0281232)
Supplement: S1 File — (DOCX) [file pone.0281232.s004.docx]

**Study protocol**

**Study population**

**Inclusion criteria**

- Adults patients aged 20–99 years with aortic stenosis

- Scheduled for elective transcatheter aortic valve implantation (TAVI) under general anesthesia via transfemoral approach at Seoul National University Hospital Cardiovascular Center, Seoul, South Korea

**Exclusion criteria**

- Non-transfemoral (i.e., transapical) approach

- Pre-procedural arterial partial pressure of oxygen (< 65 mmHg)

- Receiving oxygen treatment

- Severe pre-procedural renal dysfunction (estimated glomerular filtration rate < 30 mL/min/1.73 m^2^)

- Chronic pulmonary obstructive disease or symptomatic asthma

- Pre-procedural tuberculosis-destroyed lung

- History of lung cancer

- History of acute coronary syndrome within 6 months

- Pre-procedural elevated cardiac troponin I (cTnI) or creatine kinase-myocardial band (CKMB)

- History of stroke or transient ischemic attack within 6 months

- Pregnant

- Refuse to participate

**Enrollment and randomization**

Eligibility for TAVI was based on the consensus of a local multidisciplinary heart team, including clinical cardiologists, cardiac interventionists, cardiac surgeons, radiologists, and anesthesiologists. After enrollment and the informed consent process, the patients were randomized to receive F_I_O_2_ 0.3 or 0.8 during TAVI (1:1 allocation ratio). Block randomization (blocks of four or six) was conducted using a computer-generated program by an independent research nurse on the morning of the intervention. The group assignments were concealed in an opaque envelop, and all investigators, patients, interventionists, and data analyzers were blinded to the group allocations.

**Study protocol details**

The routine monitoring techniques of our institution for patients under general anesthesia were applied, except F_I_O_2_ management. Without premedication, 12-lead electrocardiogram, pulse oxygen saturation (SpO_2_), invasive and noninvasive arterial blood pressure, cerebral oxygen saturation (ScrbO_2_), and bispectral index monitoring were performed. Left and right ScrbO_2_ were measured using near-infrared spectroscopy (Somanetics INVOS oximeter; Covidien, Mansfield, MA, USA). Transesophageal or transthoracic echocardiography was performed to evaluate the valve position and presence of paravalvular regurgitation, as required by the interventionists.

Before inducing anesthesia, all participants were preoxygenated using an anesthesia machine (Primus; Drägerwerk, Lubeck, Germany) with F_I_O_2_ 0.3 or 0.8 according to the group allocation. After stabilization, general anesthesia was induced by a target-controlled infusion of propofol (effect-site concentration [Ce], 2.5–4.0 µg/mL) and remifentanil (Ce, 1.0–4.0 ng/mL) using a commercial infusion pump (Orchestra, Fresenius Vial, Brézins, France). Neuromuscular blockade was established by administering rocuronium (0.6 mg/kg). Then the trachea was intubated and the lungs were ventilated in volume-controlled mode with a tidal volume of 0.6–0.8 mL/kg and ventilatory rate of 9–12 /min. The alveolar recruitment maneuver was performed at 25 cmH_2_O for 10 s after tracheal intubation, and a positive end-expiratory pressure of 5 cmH_2_O was applied in all patients. According to the group assignment, F_I_O_2_ was maintained at 0.3 or 0.8 until the end of the TAVI procedure, unless the SpO_2_ was <93%. If desaturation occurred, F_I_O_2_ was increased by 0.05–0.1, and an additional alveolar recruitment maneuver was performed as needed to maintain SpO_2_ ≥93% by the attending anesthesiologists. On completing the procedure, 100% O_2_ was provided to all patients during anesthesia emergence. Patients were extubated in the intervention room, monitored in the cardiovascular care unit for 1–2 days, and then transferred to a general ward. Patients were discharged 5–7 days post-TAVI if they had no procedure-related complications.

The TAVI was conducted in accordance with the standard procedures in our institution. Using a transfemoral approach, a balloon-expandable Sapien III valve (Edwards Lifesciences, Irvine, CA, USA), self-expandable Evolut Pro or R valve (Medtronic, Minneapolis, MN, USA), or Lotus valve (Boston Scientific, Natick, MA, USA) was implanted at the diseased aortic valve. The valve was chosen by the heart team based on the size and structure of the native valve and sinus, heights of the coronary artery openings, and considerations regarding future coronary access, the risk of conduction disturbances, and annular calcification. The iliofemoral arteries were accessed under fluoroscopic guidance and closed percutaneously using Perclose ProGlide vascular suture-mediated closure devices (Abbott Vascular Devices, Redwood City, CA, USA). Before the procedure, the patients were given loading doses of dual antiplatelet agents: acetylsalicylic acid and clopidogrel (both 300 mg). During the procedure, the patients were heparinized with unfractionated heparin to achieve an activated clotting time >250 s. At completion of the valve implantation, the effects of heparin were reversed by protamine infusion.

During the procedure, arterial blood gas analysis (ABGA) was performed at four time points: baseline (before anesthesia induction, T1), after inducing general anesthesia (T2), after valve implantation (T3), and at the end of the procedure (T4). ABGA was performed using a GEM® Premier 3000 device (Model 5700; Instrumentation Laboratory, Lexington, MA, USA).

Two serum cardiac biomarkers of myocardial injury, high-sensitivity cTnI (hs-cTnI) and CK-MB, were measured at baseline (before the procedure) and 1, 4, 8, 24, 48, and 72 h after TAVI. hs-cTnI was measured using an Abbott Architect Plus Analyzer (i2000SR; Flex, San Jose, CA, USA), which has a limit of detection of 0.0011 µg/L and limit of blank of 0.0007–0.0013 µg/L. An hs-cTnI concentration ≥99^th^ percentile in the normal population (0.028 µg/L) was deemed abnormal. Serum creatinine concentrations were calibrated using isotope dilution mass spectrometry (IDMS). The eGFR was calculated using the modified diet in renal disease (MDRD) equation [1]:

IDMS MDRD eGFR = 175 × (serum creatinine)^–1.154^ × age^–0.203^ × (0.742 for women).

Postprocedural acute kidney injury (AKI) was determined based on the serum creatinine level and urine output according to the Kidney Disease: Improving Global Outcomes Clinical Practice Guidelines criteria for AKI [2]. AKI was defined as an increase in serum creatinine ≥1.5 times the baseline level or by ≥0.3 mg/dL (≥26.5 µmol/L) [AKI_creatinine_], or a urine output <0.5 mL/kg/h for ≥6 h within 7 days [AKI_urine output_]. AKI occurring >7 days after the procedure was excluded because it might have been unrelated to the procedure. Acute kidney recovery (AKR) was defined as an increase in eGFR of ≥25% relative to baseline at 48 h post-TAVI [3].

The postprocedural development of new conduction abnormalities and incidence of permanent pacemaker insertion was assessed. Stroke was defined as an acute episode of a focal or global neurological deficit as a result of hemorrhage or infarction, based on the Valve Academic Research Consortium-2 (VARC-2) definition [4]. Periprocedural MI was defined based on a combination of new ischemic symptoms or signs and elevated cardiac biomarkers within 72 h following TAVI, according to the VARC-2 definition [4].

**Study endpoints**

The primary study outcome was periprocedural myocardial injury, as reflected by the geometric area under the curve (AUC) for periprocedural serum hs-cTnI in the first 72 h post-TAVI, calculated according to the trapezoidal rule. Secondary outcomes were the AUC for serum CK-MB in the first 72 h post-TAVI, and the peak serum hs-cTnI and CK-MB levels in the same period. Post-procedural clinical outcomes were also evaluated, including AKI, AKR, new conduction abnormalities, permanent pacemaker insertion, stroke, MI, and in-hospital cardiovascular mortality.

**Sample size calculation**

We conducted a pilot study including 10 patients undergoing transfemoral TAVI under general anesthesia to calculate the sample size. The AUC for serum hs-cTnI in the first 72 h after TAVI was 40.24 ± 28.16 ng/mL. Assuming that a 50% difference in hs-cTnI levels between the two treatment groups in the first 72 h is clinically relevant, 32 patients were required for each group at 80% power and an alpha error of 5%. Considering a 10% dropout rate, we recruited 36 patients to each group (a total of 72 patients).

**Statistical analysis**

Data are presented as the mean ± SD, median (interquartile range), or number (%) after normality was tested using the Kolmogorov–Smirnov test. The primary endpoint, i.e., the AUC for serum hs-cTnI in the first 72 h after TAVI, was analyzed using the Mann–Whitney *U* test according to the data distribution. Other continuous variables were analyzed using the independent *t*-test or Mann–Whitney *U* test after performing a normality test. Categorical variables were analyzed using Pearson’s chi square test or Fisher’s exact test. For repeated measures, a linear mixed model with Bonferroni correction was used to compare the groups. In the mixed model, group, measurement time, and their interaction were fixed effects, while subject was a random effect. Plots of residuals versus fitted values were checked in terms of whether the error terms (residuals) had a mean of zero and constant variance. The normality assumption for repeated measures was assessed using histograms and quantile–quantile plots (for residuals). Multivariable logistic regression analysis was performed to identify independent risks for F_I_O_2_ and postprocedural hs-cTnI. Odds ratios were adjusted for age, sex, STS-PROM score, C-reactive protein, left ventricular ejection fraction, procedural time, and new conduction abnormality, and 95% confidence intervals were presented. The analysis was done in an intention-to-treat manner. All analyses were performed using IBM SPSS Statistics (ver. 21.0; IBM Corp., Armonk, NY, USA) or R software (ver. 3.5.1; R Development Core Team, Vienna, Austria). A P value <0.05 was considered statistically significant.

**References**

1. Levey AS, Coresh J, Greene T, Stevens LA, Zhang YL, Hendriksen S, et al. Using standardized serum creatinine values in the modification of diet in renal disease study equation for estimating glomerular filtration rate. Ann Intern Med 2006; 145: 247–254. doi: 10.7326/0003-4819-145-4-200608150-00004.

2. Khwaja A. KDIGO clinical practice guidelines for acute kidney injury. Nephron Clin Pract 2012; 120: c179­–184. doi: 10.1159/000339789.

3. Azarbal A, Malenka DJ, Huang YL, Ross CS, Solomon RJ, DeVries JT, et al. Recovery of Kidney Dysfunction After Transcatheter Aortic Valve Implantation (from the Northern New England Cardiovascular Disease Study Group). Am J Cardiol 2019; 123: 426–433. doi: 10.1016/j.amjcard.2018.10.042.

4. Kappetein AP, Head SJ, Genereux P, Piazza N, van Mieghem NM, Blackstone EH, et al. Updated standardized endpoint definitions for transcatheter aortic valve implantation: the Valve Academic Research Consortium-2 consensus document. Eur Heart J 2012; 33: 2403­–2418. doi: 10.1093/eurheartj/ehs255.
