# Supplementary material for: Effects of low versus high inspired oxygen fraction on myocardial injury after transcatheter aortic valve implantation: A randomized clinical trial
Source: PLoS One. 2023 Aug 2;18(8):e0281232. doi: 10.1371/journal.pone.0281232 (PMC10395822; doi:10.1371/journal.pone.0281232)
Supplement: S2 File — (PDF) [file pone.0281232.s005.pdf]

경피적 대동맥판막 치환술 중 산소화 정도가 심근 손상에 미치는 영향

Effect of supranormal oxygen tension on myocardial injury during transcatheter aortic valve replacement

Version No: 1.5

책임연구자 소속: 서울대학교병원 마취통증의학과
책임연구자 이름: 전 윤 석

연구 개요

연구제목	(국문) 경피적 대동맥판막 치환술 중 산소화 정도가 심근 손상에 미치는 영향
	(영문) Effect of supranormal oxygen tension on myocardial injury during transcatheter aortic valve replacement
책임연구자	마취통증의학과 교수 전윤석
연구비 지원기관	없음
연구 목적	경피적 대동맥판막 치환술을 위한 마취 중 산소화 정도에 따라 시술 후 심근 손상 지표에 차이가 있는지 알아보고자 한다.
연구 설계	무작위 배정 임상시험
연구 기간	IRB승인일 ~ 60개월
연구 대상	경피적 대동맥판막 치환술을 마취 하에 받는 성인 환자
연구 대상자 수	총 72명
취약한 연구대상자	해당 없음
연구 방법	경피적 대동맥판막 치환술을 받는 환자를 정상산소혈증군과 고산소혈증군에 무작위 배정하여, 전신마취 유도 시부터 기계환기기의 흡입산소분율을 정상산소혈증은 0.3, 고산소혈증은 0.8로 유지하고 시술 중 맥박산소포화도를 최소 93% 이상 유지하도록 흡입산소분율을 조절한다.<br>시술 후 3일 동안 두 군간 serum troponin I을 측정하여 area under the curve (AUC)을 비교하여 마취중 산소화에 따른 심근 손상의 정도를 비교한다.
유효성 평가	본 연구를 통하여 경피적 대동맥판막 치환술을 받는 환자의 임상 관리에서 심근 등 주요 장기의 손상을 최소화하며 임상적 예후를 개선시킬 수 있는 적절한 산소 공급의 지표에 대한 이론적 근거를 마련할 수 있을 것이다.
안전성 평가	본 연구에서는 통상적인 경우와 마찬가지로 동맥산소분압 및 산소포화도를 확인하며 산소 공급을 시행하므로 저산소증의 위험을 최소화하여 연구를 수행하는 것이 가능하며, 또한 예상치 않은 지속적 저산소증이 발생할 경우 연구를 중단하고 필요한 조치를 취하게 되므로 대상자에게 가해질 위험성은 최소라고 할 수 있다.
기대효과 및 예상결과	대동맥판막 질환자에서 경피적 판막 치환술 중 적절한 산소 공급의 지표를 확립하여 대동맥판막 질환자의 판막 시술 등 임상 관리에서 심근 등 주요 장기의 손상을 최소화하며 임상적 예후를 개선시킬 수 있는 적절한 산소화 정도의 기준을 제공하고 그 이론적 근거를 마련할 수 있을 것이다.

연구계획서

1. 연구 제목
경피적 대동맥판막 치환술 중 산소화 정도가 심근 손상에 미치는 영향
(Effect of supranormal oxygen tension on myocardial injury during transcatheter aortic valve replacement)

2. 연구 실시기관 명칭 및 주소
서울대학교병원, 서울시 종로구 대학로 101

3. 연구책임자 및 공동연구자 성명 및 직명
1) **연구책임자:** 전윤석 (교수)
2) **공동연구자:** 김효수 (교수), 남가람 (임상강사)
3) **연구담당자:** 조연정 (진료교수)
4) **임상시험용 의약품 관리약사 / 임상시험용 의료기기 관리자:** 해당 없음

4. 연구 의뢰기관
1) **연구 의뢰기관 명칭 및 주소:** 해당 없음
2) **모니터요원 성명 및 직명:** 서정화 (부교수)

5. 연구비 지원기관 명칭 및 주소
해당없음

6. 예상연구기간
승인일로부터 60개월

7. 연구 대상 질환
경피적 대동맥판막 치환술의 적응증이 되는 대동맥판막 협착증

8. 연구의 배경 및 목적
1) 연구 배경
심장 질환 환자의 경피적 판막 치환술 등 마취가 필요한 시술 중에는 조직의 저산소증을 예방하기 위해 관례적으로 O_2 reservoir를 확보하는 차원에서 마취 중 추가적인 산소(routine use of supplemental oxygen)를 공급하고 있는데, 이로 인하여 의도하지 않은 고산소혈증이 종종 발생할 수 있다. 그러나 필요 이상으로 높은 산소화(hyperoxygenation)는 산화 스트레스(oxidative stress), 혈관 수축(vasoconstriction), 그리고 관류의 비균질성

(perfusion heterogeneity)을 유발하여 이로 인한 심근 손상(myocardial injury)의 가능성이 있고 임상적 예후(clinical outcome)에도 영향을 줄 수 있는 것으로 알려져 있다.[1-3] 기존 연구에서 cardiac arrest에서 resuscitation 된 후 동맥산소분압이 높을 수록 재원기간 중 사망률이 증가하였다는 연구 결과가 있고,[4] 급성 심근경색 환자에서 의례적으로 산소를 공급하는 것이 결정적 근거가 부족하다는 systematic review의 보고가 있었지만,[5] 경피적 대동맥판막 치환술을 받는 환자에서 산소화 정도에 따른 주요 장기 손상에 대한 데이터나 임상적 예후와 관련해서는 아직까지 그 임상적 근거가 부족한 실정이어서 무작위 배정 등의 임상 연구가 더 필요한 상황이다.

2) 연구 가설 및 목적

본 연구의 가설은 경피적 대동맥판막 치환술 중 정상산소혈증이 유지된 군은 과산소혈증이 유지된 군에 비해 수술 후 area under the time-to-troponin I curve (TnI AUC) 로 측정한 심근손상의 지표가 낮을 것이라는 것이다. 본 연구 결과 대동맥판막 질환자에서 경피적 판막 치환술 중 적절한 산소 공급의 지표가 확립되면 대동맥판막 질환자의 판막 시술 등 임상 관리에서 심근 등 주요 장기의 손상을 최소화하며 임상적 예후를 개선시킬 수 있는 적절한 산소화 정도의 기준을 제공할 수 있을 것이다.

9. 시험용 의약품의 코드명이나 주성분의 일반명, 원료약품 및 그 분량, 제형 등

본 연구에서는 경피적 대동맥판막 치환술을 받는 환자의 마취 중 산소화 정도를 양 군에 다르게 적용시키는 연구로, 공급되는 산소의 흡입분율 (FiO_2)에 차이가 있다.

시험용 중재 대상(수술법, 진단법 등)

대동맥판막 협착증 환자 중 마취 하 경피적 대동맥판막 치환술을 받는 환자를 대상으로 한다.

10. 연구대상자의 선정 기준, 제외 기준, 목표한 대상자 수 및 산출 근거

1) 선정기준

- 나이 20세 이상 성인
- 대동맥판막 협착증으로 경피적 대동맥판막 치환술을 받기로 예정된 환자
- 성별 및 인종 제한 없음

2) 제외기준

- myocardium에 직접 손상을 줄 수 있는 transapical approach로 경피적 대동맥판막 치환술을 시행하는 경우
- 시술 전 동맥혈가스분석 상 동맥산소분압이 65 mmHg 미만이거나 시술 전oxygen supply 또는 기계 환기를 받고 있는 경우
- 시술 전 심각한 신기능 이상(end-stage renal disease)이 있는 경우
- 시술 전 만성 폐쇄성 폐질환, 천식, 간질성 폐질환, (chronic obstrucrtive lung

disease, asthma, interstitial lung disease)
- 시술 전 Tb-destroyed lung
- 시술 전 history of lung cancer
- History of acute coronary syndrome (unstable angina, acute myocardial infarction) within the past 6 months
- 시술 전 elevated troponin I or CK-MB
- 시술 6개월 이내 stroke이나 transient ischemic attack이 있었던 경우
- 연구 참여에 동의하지 않은 경우
- pregnant

3) 목표한 대상자 수 및 산출 근거

경피적 대동맥판막 치환술을 받는 환자의 마취 중 정상산소혈증이 유지된 군과 고산소혈증이 유지된 군 간 심근 손상 등 주요 장기에 미치는 영향을 비교하기 위해 연구의 일차임상종점을 시술 후 0, 4, 8, 24, 48, 72 시간 째 측정한 serum troponin I level의 area under the curve (TnI AUC)의 군 간 차이로 하였다. 경피적 대동맥판막 치환술 후 TnI AUC 를 다룬 선행 연구가 없어, 서울대학교병원에서 전신마취 하 경피적 대동맥판막 치환술을 시행받은 환자 10명의 검사 기록을 통해 TnI AUC를 trapezoidal method로 계산하였을 때 평균 40.24, 표준편차 28.16 이었다. 정상산소혈증이 유지된 군에서 고산소혈증 군보다 TnI AUC가 50% 감소하면 의미있다고 가정하고, 두 군의 차이를 independent *t*-test로 비교할 때, α 오류 0.05, β 오류 0.2로 제한하면, 필요한 대상자 수는 군 당 32명이며, 10%의 탈락률을 고려하면 각 군 당 36명으로 총 72명의 대상자 모집이 필요하다.

4) 연구 대상자 모집 계획

서울대학교병원에서 경피적 대동맥판막 치환술을 받기로 예정된 환자를 대상으로 선정기준과 제외기준에 만족하는지 확인한 후 시술 전 대상자를 직접 방문하여 연구 목적, 참여 방법, 연구 절차, 대상자에게 예상되는 이익과 손해, 연구 참여에 따르는 보상(보상 없음), 연구에 참여하지 않았을 때 예상되는 불이익(불이익 없음), 연구에 쓰이는 중재 방법(마취 중 흡입산소분율/산소유량의 차이) 및 예상되는 부작용, 선택 가능한 대체법 등에 대해 비의료인이 이해하기 쉬운 용어로 설명한 후 충분한 설명에 근거한 자발적 동의를 정해진 동의서에 서면으로 얻어 대상자를 모집한다.

본 연구의 책임연구자 및 공동연구자는 인종이나 사회경제적 상태에만 근거해서 이 연구에 참여할 가능성이 있는 대상자를 배제시키지 않을 것이며, 연구의 선정 기준에 합당하다면, 가능한 대상자가 이 연구에 참여할 수 있도록 노력할 것이며, 본 기관에서 치료받는 판막 질환자의 전체를 대표할 수 있도록 대상자에게 연구의 목적을 주지시킬 것이다.

11. 연구 방법

1) 구체적인 연구방법

연구에 참여하기로 동의한 대상자는 본 연구와 상관없이 통상적으로 시행되는 절차에 따라 시술 전 준비 과정을 거쳐 시술장에 입실한다. 비침습적 혈압, 5극 심전도, 심전도 및 심박수 변이, 맥박산소포화도, bispectral index (BIS)를 통한 마취심도, 그리고 forehead에서 cerebral oximeter를 감시하면서 radial artery에 arterial cannula를 거치하여 지속적 침습 동맥압을 감시한다. 마취 유도 및 유지는 propofol, remifentanil의 목표농도주입법으로 전정맥마취를 이용하며, BIS (bispectral index) 값이 60 이하로 유지되도록 마취 심도를 조절한다.

전신마취 유도 시부터 기계환기기의 흡입산소분율을 정상산소혈증군은 0.3, 고산소혈증은 0.8로 유지하고 시술 중 맥박산소포화도를 최소 93% 이상 유지하도록 흡입산소분율을 조절한다.

두 군 모두에서 기관삽관 직후 alveolar recruitment maneuver (폐포동원술, 25 cmH_2O의 양압으로 10초 간 폐포 확장)를 시행 후 positive end-expiratory pressure (PEEP) 5 cmH_2O를 적용하여 전신마취에 의한 atelectasis를 예방하고 적절한 산소화가 유지되도록 돕는다.

시술이 끝나면 통상적인 방법대로 마취에서 환자를 회복시켜 심혈관계 집중관찰실에서 활력징후를 관찰 후 일반 병실로 전동하도록 한다.

시술 중 동맥산소분압은 마취 유도 전 (T0, baseline), 마취유도 10분 후 (T1), valve implantation 후 (T2), 시술이 끝나는 시점 (T3)에 측정한다. 동맥혈산소분압 결과에 따라 흡입산소분율을 조정할 경우, 조정 후 10분 뒤 동맥산소분압 결과를 다시 확인한다. Serum troponin I는 시술 직후, 시술 후 4/8/24/48/72시간에 측정한다.

2) 비교군 설정 및 무작위 배정 방법

연구에 참여하지 않는 제 3자를 통해 무작위 배정 컴퓨터 프로그램(Random Allocation Software, version 1.0)을 이용하여 정상산소혈증군과 고산소혈증군에 1:1로 배정되도록 블록크기 4 또는 6의 무작위 배정표를 생성하고 보관한다. 연구 대상자로 선정된 후 이 배정표에 따라 대상자를 정상산소혈증군 또는 고산소혈증군에 배정한다. 무작위 배정표는 대상자가 연구 대상자로 선정되기 전에 연구에 참여하는 대상자를 볼 수 없고 연구와 독립되어 있는 제 3자에 의해 생성, 운영, 관리되어 allocation concealment를 유지하도록 한다. 연구 대상자는 마취 전 대상자가 어느 군에 속하여 어떤 흡입산소분율을 사용할 지 사전에 알 수 없게 하며 시술 중 적용되는 산소분율 또한 대상자가 마취되어 있는 동안 투여하게 되므로 대상자가 알 수 없게 된다. 마취를 시행하는 사람과 각종 데이터를 수집, 기록, 분석하는 사람을 분리시켜 각 데이터가 어느 군에 속한 대상자로부터 얻어진 것인지 알 수 없게 한다. 눈가림 해지는 연구 관련

데이터 수집이 모두 끝난 후에 연구 결과 분석에 영향이 없음을 확인한 후 눈가림 해지를 하도록 한다.

3) 시험 중재에 대한 설명

본 연구는 시술을 위한 마취 중 제공되는 흡입산소분율 및 산소유량을 다르게 하여 산소화정도에 따라 시술 후 심근 손상 지표에 차이가 있는지 알아보고자 하는 연구이다. 무작위 배정된 군에 따라 전신마취 유도 시부터 기계환기기의 흡입산소분율을 정상산소혈증은 0.3, 고산소혈증은 0.8로 유지하고 시술 중 맥박산소포화도를 최소 93% 이상 유지하도록 흡입산소분율을 조절한다. 두 군 모두에서 기관삽관 직후 alveolar recruitment maneuver (폐포동원술, 25 cmH_2O pressure로 10초간 폐포 확장)를 시행 후 positive end-expiratory pressure (PEEP) 5 cmH_2O를 적용하여 전신마취에 의한 atelectasis를 예방하고 시술 중 적절한 산소화가 유지되도록 돕는다.

시술 중 동맥산소분압은 마취 유도 전 (T0, baseline), 마취유도 10분 후 (T1), valve implantation 후 (T2), 시술이 끝나는 시점 (T3)에 측정한다. 흡입산소분율을 조정할 경우, 조정 후 10분 뒤 동맥혈가스검사 (ABGA) 결과를 다시 확인한다. Serum troponin I는 시술 직후, 시술 후 4/8/24/48/72시간에 측정한다.

4) 관찰항목, 임상검사항목 및 관찰검사방법

본 연구의 일차임상종점은 시술 후 3일 간 측정한 serum troponin I의 area under curve (TnI AUC)이다. 이차임상종점은 시술 중 동맥산소분압 측정 시 동시에 측정한 cerebral oximeter의 변화, 시술 후 3일 간 측정한 CK-MB, 입원기간 중 새롭게 발생한 delirium, acute kidney injury 혹은 renal replacement therapy, stroke, myocardial infarction, mortality의 발생, 시술 후 집중관찰실 재원기간, 그리고 시술 후 병원 재원기간으로 한다.

연구 대상자로부터 수집하는 기본적인 임상적 특징은 다음과 같다.
- 성별, 나이, 신장, 체중, 체질량지수, 체표면적
- 시술 전 진단명, 시술명, 시술 후 진단명
- 기저 질환, 흡연력, 투약력, 시술 전 지속 정주 약제의 종류 및 용량
- 시술 전후 시행한 검사 (혈액 검체, 영상, 기능 검사) 결과
- 시술 전후 동맥산소분압, CK-MB, cardiac troponin I 수치

시술 및 마취 중 관찰 및 시행하는 검사 항목은 다음과 같다.
- 혈압, 심전도, 심박수, 심박수 변이, 산소포화도, 중심정맥압, 폐동맥압, 체온
- Bispectral index, cerebral oximeter, mixed venous oxygen saturation
- Cardiac index/output, stroke volume variation, oxygen reserve index

- 각 마취제의 농도, 흡입산소분율, 산소 유량, 조직산소포화도
- 시술 중 주입된 수액 및 수혈 양
- Rotational thromboelastometry
- ACT, Coagulation test, Multiplate platelet function analysis
- 시술시 사용된 헤파린 양, 시술 시간, 마취 시간
- 시술 중 추정 실혈양, 배뇨량
- 시술 중, 후 심혈관계 약제의 사용량
- 시술 후 산소요법 경로 및 양
- 중환자실 혹은 집중관찰실 체류 기간, 시술후 재원 기간, 기계환기 시간
- 새롭게 발생한 delirium, acute kidney injury, renal replacement therapy, stroke, myocardial infarction, mortality 등 주술기 주요 심혈관 및 뇌혈관 합병증 발생
- 기타 특이 사항

5) 기존 치료 및 연구와의 차별점

대동맥판막 협착증 환자를 대상으로 경피적 대동맥판막 치환술을 시행하는 치료 과정은 동일하나, 마취 과정 중 기존에 불필요하게 고산소혈증을 유발할 수 있었던 기존 관리 방법과 비교하여 정상산소혈증을 유지할 때 고산소혈증으로 유발 가능한 심근 손상을 줄이고 환자의 임상적 예후를 개선시킬 수 있을지 알아보아 적절하고 안전한 환자 마취 관리에 그 이론적 근거를 마련하고자 한다. 기존에 cardiac arrest 환자에서 지나친 산소화로 인한 사망률의 증가에 대한 보고나[4] 급성 심근경색 환자에서 관례적으로 공급하는 산소 치료에 결론적인 근거가 부족하다는 systematic review는 있었지만,[5] 경피적 대동맥판막 치환술을 받는 환자에서는 이와 관련한 연구가 부족하여 무작위 배정 임상 시험이 필요한 상황이다.

6) 연구대상자의 이익과 위험

본 연구에서는 통상적인 경우와 마찬가지로 동맥산소분압 및 산소포화도를 확인하며 산소 공급을 시행하므로 저산소증의 위험을 최소화하여 연구를 수행하는 것이 가능하며, 또한 예상치 않은 지속적 저산소증이 발생할 경우 연구를 중단하고 필요한 조치를 취하게 되므로 대상자에게 가해질 위험성을 최소화할 수 있다.

본 연구에서 동맥산소분압 및 심근 손상의 지표를 측정하기 위해 시행되는 검사는 본 연구와 상관 없이 통상적인 대동맥판막 치환술 환자에서 시술 후 시행되는 검사의 종류 및 범위와 다르지 않으며, 본 연구를 위해 추가적으로 시행되는 검사 비용에 대해서는 대상자가 부담하도록 하지 않는다.

7) 중지·탈락 기준

연구 참여 동의 후에라도 대상자가 동의를 철회하거나 연구 참여를 거부하는 경우 연구를 중지하고 대상자에서 제외시키기로 한다. 시술 중 예상치 못한 이벤트로 인하여 심각한 저산소증이 지속(동맥산소분압 60 mmHg 미만 혹은 맥박산소포화도 80% 미만으로 30분 이상 지속)되거나 연구를 지속하기 어렵다고 판단될 경우에는 연구 수행을 중단하고 필요한 조치를 취하도록 한다. 연구 프로토콜을 중단한 대상자에 대해서는 따로 보고하도록 하고, 최종 분석에서는 제외하도록 한다.

8) 부작용을 포함한 안전성의 평가기준, 평가 방법 및 보고 방법

본 연구에 참여한 모든 환자를 대상으로 안정성 평가를 실시한다. 연구 시행 중에 이상 반응이 발생했을 경우, 연구에 사용된 시험 방법과의 인과 관계 유무와 모든 이상 반응을 기록하고 추후 중증도, 중대성, 기간, 그리고 시험 방법과의 인과 관계를 평가한다. 이상 반응에 대한 처치 및 결과 역시 기록한다. 이상 반응은 시험 기간 중의 계획된 검진과 검사의 소견 이외에도 비정상적인 검진이나 필요에 따른 추가적인 검사와 검진에 의해 평가하고 즉각적인 조치를 시행한다. 임상병리검사 자료에 대해서는 변수의 특성에 따라 치료 전, 후의 군내 비교 등 적절한 통계 방법을 이용하여 분석하고, 이상 반응의 빈도, 발현율, 각각의 목록, 심각한 정도 및 시험 방법과의 인과 관계 등을 제시하며, 필요한 경우 그래프 형태로 보고한다.

(1) 중증도

이상 반응은 아래의 정의에 따라 경증, 중등증, 중증으로 구분한다.
경증은 일반적이고 일시적이고, 일상적인 활동을 방해하지 않는다.
중등증은 약간의 불편함을 초래하거나 일상적인 활동을 방해한다.
중증은 일상적인 활동을 수행할 수 없다.

(2) 인과 관계

인과 관계는 ‘관련 없을 것으로 생각됨’, ‘관련 있을 가능성 있음’, ’가능성 많음’, ’명백히 관련 있음’, 또는 ‘관련성을 확인하기 어려움’으로 구분된다.

9) 효과 평가기준, 평가 방법 및 해석방법 (통계분석방법 등)

본 연구의 일차임상종점은 시술 후 3일 간 측정한 serum troponin I level의 area under the curve (TnI AUC)이다. 두 군의 비교 시 분석군은 일차적으로 intention-to-treat manner로 정의하고, per-protocol, as-treated manner로도 추가 분석한다.

두 군 간 TnI AUC에 차이가 있는지 검정하기 위해 각 군의 평균값을 independent *t*-test를 이용하여 비교하고, p값이 0.05 미만인 경우 통계적으로 유의한 차이가 있다고 판단한다.

이차임상종점은 시술 후 입원기간 동안 시술 중 동맥산소분압 측정 시 동시에 측정한 cerebral oximeter의 변화, 시술 후 3일 간 측정한 CK-MB, 입원기간 중 새롭게 발생한 delirium (CAM-ICU[6] 을 이용하여 attending nurse가 daily basis로 평가함 ➔ 특성

1: 급성으로 발생하였거나 계속 변화하는 경과인가?, 특성 2: 주의력 결핍, 특성 3: 의식 수준의 변화, 특성 4: 비체계적인 사고, 최종 양/음성 여부는 특성 1과 2, 그리고 특성 3 또는 4가 존재할 경우 양성으로 판단함), acute kidney injury 혹은 renal replacement therapy, stroke, myocardial infarction, mortality의 발생, 시술 후 집중 관찰실 재원기간, 그리고 시술 후 병원 재원기간으로 한다. 이 외 대상자의 기본 임상 정보 및 혈압, 심박수, 심박수 변이, 산소포화도, oxygen reserve index, 동맥산소분압, 각종 검사 및 임상 수치들에 대하여 연속변수인 경우 평균과 표준편차 또는 중앙값과 25-75 퍼센타일로 표시하고 두 군간 차이는 independent *t*-test 또는 Mann-Whitney U test로 비교하며, 비연속변수인 경우에는 빈도수와 퍼센트로 표시하고 두 군간 차이는 Chi-square test, Fisher's exact test로 비교한다. 여러 시점에 측정된 값의 비교는 RMANOVA 또는 linear mixed model을 이용하여 분석한다. 통계 분석은 윈도우용 SPSS (ver 22; IBM corp., Armonk, NY, USA) 및 R software (ver 3.4.0; R Core Development Team, Vienna, Austria)를 사용한다.

10) 임상시험 후 연구대상자의 진료 및 치료기준

임상 시험 후 연구대상자의 진료 및 치료는 경피적 대동맥판막 치환술 환자의 통상적인 시술 후 진료 및 치료 기준에 따르도록 한다. 본 연구와 관련하여 문의 사항이 있을 경우 연락할 수 있는 연구자의 연락처를 제공하도록 한다.

11) 연구수행일정표

승인일로부터 7개월: 연구 대상자 모집 및 연구 시행

승인일로부터 8~12개월: 자료 분석 및 논문 집필

12. 자료 및 안전성 모니터링 계획 (Data and Safety Monitoring Plan)

1) 모니터링 책임자

***모니터링 책임자: 전윤석**

***모니터링 담당자: 서정화**

2) 자료 및 안전성 정보 모니터링 항목

*자료(study accruals) 항목: 혈압, 맥박수, 산소포화도, 동맥산소분압 등 연구 대상자 정보와 관련하여 보호되어야 할 자료

*안전성(Safety) 항목: 산소 공급에 따른 동맥산소분압의 변화와 시술 후 심근 손상 지표값의 변화

3) 자료 및 안전성 모니터링 방법 및 주기

모든 연구 대상자에 대하여 지속적인 안전성 모니터링을 실시한다. 안전성 모니터링은 연구에 관여하지 않는 제3자가 하도록 한다. 연구의 지속, 변경, 중단을 결정할 수

있는 사안, 주요 유효성 평가 변수에 대한 모니터링을 실시한다. 모니터링 주기는 6개월 마다 시행하기로 하고, 필요에 따라 모니터링이 더 자주 필요하게 되는 사안이 생길 경우 주기를 조정하도록 한다.

본 연구에서는 통상적인 경우와 마찬가지로 동맥산소분압 및 산소포화도를 주기적으로 확인하며 산소 공급을 조절하므로 연구 참여 여부와 무관하게 저산소증의 큰 위험 없이 안전하게 연구를 수행하는 것이 가능하며, 또한 예상치 않은 지속적 저산소증이 발생할 경우 연구를 중단하고 필요한 조치를 취하게 되므로 대상자에게 가해질 위험성은 최소라고 할 수 있다.

4) 이상약물반응보고, 연구 미준수, 예상하지 못한 문제의 보고

모니터링 결과에 따라 해당하는 경우 필요에 따라 IRB 또는 해당 규제 기관에 절차에 따라 보고하도록 한다. 이상 반응, 예상하지 못한 문제, 계획서 미준수 등이 발생했을 경우 정해진 기한 안에 절차에 따라 해당 기관에 보고하도록 한다. 본원에서 발생한 중대한 이상약물반응, 중대한 연구 미준수, 예상하지 못한 문제가 발생한 경우 연구자가 인지한 날로부터 근무일 15일 이내에 IRB에 보고하도록 한다.

5) 연구 중단 기준

연구 참여 동의 후에라도 대상자가 동의를 철회하거나 연구 참여를 거부하는 경우 연구를 중지하고 대상자에서 제외시키기로 한다. 시술 중 예상치 못한 이벤트로 인하여 심각한 저산소증이 지속(동맥산소분압 60 mmHg 미만 혹은 맥박산소포화도 80% 미만으로 30분 이상 지속)되거나 연구를 지속하기 어렵다고 판단될 경우에는 연구 수행을 중단하고 필요한 조치를 취하도록 한다. 연구 프로토콜을 중단한 대상자에 대해서는 따로 보고하도록 하고, 최종 분석에서는 제외하도록 한다. 목표한 연구 자료를 모두 획득하였거나 또는 연구 종점에 도달할 수 없다고 판단될 때에는 연구의 지속, 중단(stop)이나 변경(alteration) 등을 결정하도록 한다. 연구를 중단하게 될 때에는, 연구를 중단하게 된 배경과 예상되는 원인을 파악하여 이를 개선할 수 있는 방안을 모색하여 IRB에 보고하도록 하고, 후속 조치 및 후속 연구 계획에 대한 사항을 함께 보고하고 시행하도록 한다.

13. 연구대상자의 안전보호를 위한 대책

1) 연구의 윤리성 확보를 위한 기본 방안

본 연구의 계획과 수행에 있어 가장 최신의 헬싱키 선언(2013년 개정) 및 ICH-GCP를 준수할 것이며, IRB의 승인 절차 후 대상자 모집 및 동의서 취득을 포함한 모든 연구 절차를 수행할 것이다. 피험자 안내문 및 동의서에 연구 과정과 관련하여 의문 사항이나 불편 사항이 있을 경우 연락할 수 있는 담당자의 이름과 연락처를 명시하도록 한

다.

연구 계획서에 명시되지 않은 윤리적 사항은 세계 의학 연합체의 헬싱키 선언에 기반을 두고 집행될 것이다. 또한, 연구 수행 중 헬싱키 선언에 피험자 보호 사항에 저촉될 가능성이 있는 경우에는 연구를 중단하고, 의학연구윤리심의 위원회에 보고할 예정이다. 본 연구는 병원윤리위원회의 윤리규정을 준수할 것이다.

2) 연구대상자의 동의 과정

연구 대상자 선정 후 대상자에게 연구에 대한 설명, 동의를 취득할 연구자는 책임연구자, 공동연구자, 연구담당자로 한한다. 동의를 제공할 수 있는 자는 연구 대상자 본인으로 한다. 연구 설명 과정과 동의 취득 과정 중 대기 시간은 대상자가 연구에 대한 설명을 들은 후 충분한 시간을 갖고 동의하고자 결정할 때까지로 한다. 이 때 대상자가 동의를 결정하기까지 오랜 시일이 걸리는 경우 동의 전 모집하고자 하는 모든 대상자가 모집된 경우 연구 참여가 제한될 수 있음을 설명한다. 대상자 스스로 동의를 결정하기까지 연구 참여를 강제하거나 연구 참여 결정에 부당한 영향을 미칠 수 있는 정보를 강요하지 않도록 한다. 연구 설명 과정과 동의 취득 과정 중에 사용되는 언어는 연구대상자가 이해할 수 있는 언어를 사용하며, 설명이 필요한 부분에 대해 동의 제공자는 설명을 요구할 수 있고 연구자는 충분한 설명을 제공하도록 한다. 강제 또는 부당한 영향의 가능성을 최소화 하기 위해 서면 동의서 및 구두 설명 과정에서 연구에 참여하지 않음으로 받는 불이익이 없음과 연구 참여로 인해 취득할 수 있는 직접적 이익이 없음을 명시하도록 한다. 연구 대상자에게 제공하는 정보와 동의서 형식은 본 연구계획서에 별첨하도록 한다.

3) 연구대상자의 보상 방안

본 연구에 참여함으로써 대상자가 제공받는 사례나 직접적 보상은 없으며, 이를 대상자에게 사전에 고지한다.

4) 연구대상자의 개인정보보호 방안

연구 대상자, 연구자, 연구 시험 참여자 이외에는 대상자의 시험 참여 여부나 경과에 대해 알지 못하게 하며, 연구 대상자의 신원을 파악할 수 있는 기록은 비밀로 보장될 것이다. 대상자의 관리 번호 등은 주관연구자의 책임하에 별도의 파일로 보관하며 이를 코드화하여 연구데이터를 통하여 개인 신상 확인이 불가능하도록 관리한다. 연구를 위해 수집되는 정보는 잠금 장치가 있는 연구실에 비밀번호가 걸린 파일로 보관하고 연구 파일에 접근할 수 있는 사람은 권한을 가진 일부 연구자로 제한할 것이다. 수집되는 자료의 불필요한 개인식별자는 제거하고, 특히, 증례기록서에는 대상자의 이름, 주민등록번호, 차트 번호 등을 기재하지 않도록 하며, 신상 정보와 연결된 식별자 코드는 별도로 관리할 것이다. (환자와 관련된 사진을 제출할 때는 환자의 신원을 알 수 없도록 할

것이며 조금이라도 신원이 노출될 가능성이 있는 경우에는 이에 대한 서면 동의를 받았음을 명시할 것이다.) 대상자에 대한 기록은 비밀이 유지되고 다른 곳으로 이동되지 않을 것이며, 본 연구의 진행 여부를 감독 받기 위해 감독 기관으로 보내어질 수 있다. 생명윤리법 시행규칙 제15조에 따라 연구 관련 기록을 연구가 종료된 시점부터 3년간 보관하도록 하며, 보관기관이 지난 문서 중 개인정보에 관한 사항은 개인정보보호법 시행령 제16조에 따라 파기하도록 한다. 책임연구자와 공동연구자가 정기적으로 시험 기관을 방문하여 대상자 모집, 대상자 등록, 데이터 저장 및 분석에 대한 평가, 임상시험계획서와 GCP의 준수 여부를 확인할 것이다.

본 연구의 목적으로 수집된 정보는 어떠한 목적으로도 제3자에게 제공될 계획이 없으며, 본 연구에서는 유전자 정보 혹은 가계도 정보를 포함하지 않을 것이다.

5) 취약한 연구대상자를 포함하는 경우 추가적인 보호조치 방안

본 연구는 미성년자, 임산부, 태아, 신생아, 손상된 동의 능력을 가진 성인, 학생, 피고용자, 수감자 등 취약한 연구대상자를 포함하지 않는다.

14. 인체유래물의 보관 및 폐기 방법

대상자로부터 얻은 혈액 검체 등의 인체유래물은 본 연구에 사용되는 목적 이외에는 따로 보관되지 않으며, 본 연구를 위한 목적(동맥산소분압, cardiac enzymes의 측정 등)을 위한 분석 후 폐기한다.

15. 참고 문헌

1. Spoelstra-de Man AM, Smit B, Oudemans-van Straaten HM, Smulders YM. Cardiovascular effects of hyperoxia during and after cardiac surgery. Anaesthesia 2015;70:1307-19.
2. Paparella D, Guida P, Caparrotti S, et al. Myocardial damage influences short- and mid-term survival after valve surgery: a prospective multicenter study. J Thorac Cardiovasc Surg 2014;148:2373-9 e1.
3. Bouleti C, Chauvet M, Franchineau G, et al. The impact of the development of transcatheter aortic valve implantation on the management of severe aortic stenosis in high-risk patients: treatment strategies and outcome. Eur J Cardiothorac Surg 2017;51:80-8.
4. Kilgannon JH, Jones AE, Parrillo JE, et al. Relationship between supranormal oxygen tension and outcome after resuscitation from cardiac arrest. Circulation 2011;123:2717-22.
5. Cabello JB, Burls A, Emparanza JI, Bayliss S, Quinn T. Oxygen therapy for acute myocardial infarction. Cochrane Database Syst Rev 2013:CD007160.

6. Ely EW, Inouye SK, Bernard GR, et al. Delirium in mechanically ventilated patients: validity and reliability of the confusion assessment method for the intensive care unit (CAM-ICU). JAMA 2001;286:2703-10.

[별첨 1] 연구대상자 설명문 및 동의서

대상자 일련번호	

연구대상자 설명문

1. 임상연구 제목: 경피적 대동맥판막 치환술 중 산소화 정도가 심근 손상에 미치는 영향

2. 연구책임자: 마취통증의학과 교수 전윤석

3. 임상연구의 배경 및 목적: 심장 질환 환자의 경피적 판막 치환술 등 마취가 필요한 시술 중에는 조직의 저산소증을 예방하기 위해 관례적으로 추가적인 산소를 공급하게 되어 의도하지 않은 고산소혈증이 종종 발생할 수 있습니다. 그러나 필요 이상으로 높은 산소화는 산화 스트레스, 혈관 수축 등을 유발하여 이로 인한 심장 근육의 손상의 가능성이 있고 예후에도 영향을 줄 수 있는 것으로 알려져 있으나, 경피적 대동맥판막 치환술을 받는 환자에서 산소화 정도에 따른 주요 장기 손상에 대한 데이터나 그 임상적 근거가 부족한 실정이어서 무작위 배정 등의 임상 연구가 필요한 상황입니다.

4. 임상연구 참여대상자 수 및 참여기간: 경피적 대동맥판막 치환술을 받는 환자분 총 72명을 대상으로 정상산소혈증군과 고산소혈증군에 웹프로그램을 이용하여 1:1로 무작위 배정 ('동전던지기'와 같이 우연에 의해 배정)되어 연구가 이루어지며, 무작위 배정의 결과는 별도로 관리되어 연구 진행 전 및 연구 중에 연구진 및 참여대상 환자가 알 수 없도록 합니다. 연구 참여 기간은 시술 중 마취를 받는 기간으로, 시술 후에는 통상적인 진료 과정을 거치게 됩니다.

5. 임상연구의 절차 및 방법

연구에 참여하기로 동의한 대상자는 통상적으로 시행되는 절차에 따라 시술 전 준비 과정을 거쳐 시술장에 입실하게 됩니다. 마취 유도 및 유지는 프로포폴과 (마약성 진통제의 일종인) 레미펜타닐을 이용한 정맥마취를 받게 되며 뇌파를 이용한 장비를 통한 감시로 마취 심도를 조절하게 됩니다. 전신마취 유도시부터 기계환기기의 흡입산소분율을 정상산소혈증은 0.3, 고산소혈증은 0.8로 유지하고 시술 중 맥박산소포화도를 최소 93% 이상 유지하도록 흡입산소분율을 조절합니다. 두 군 모두에서 기관삽관 직후 폐포동원술을 시행하여 전신마취에 의한 폐포의 허탈을 방지하고 적절한 산소화가 유지되도록 돕습니다.

시술이 끝나면 통상적인 방법대로 마취에서 환자를 회복시켜 심혈관계 집중관찰실에서 활력징후를 관찰 후 일반 병실로 전동하도록 하여 통상적인 진료 과정을 거치게 됩니다.

시술 중 동맥산소분압은 마취 유도 전, 마취 유도 10분 후, 판막 거치 후, 시술이 끝나기 전에 각각 측정하며, 심근 손상의 지표(troponin I)는 시술 전, 시술 직후, 시술 4/8시간 후, 시술 후 1, 2, 3일 째에 각각 측정하게 됩니다.

본 연구 과정은 대상자가 마취를 받는 과정 중 산소를 공급받는 정도에 관한 연구로 연구 대상자분이 준수해야 할 사항은 없습니다.

6. 연구대상자에게 예견되는 부작용, 위험과 불편함

본 연구는 유효성 및 효과가 검증되지 않은 임상 시험으로, 통상적인 경우와 마찬가지로 동맥산소분압 및 맥박산소포화도를 주기적으로 확인하며 산소 공급을 시행하므로 연구 참여 여부와 관계없이 저산소증의 위험 없이 안전하게 연구를 수행하는 것이 가능하며, 예상치 않은 지속적 저산소증이 발생할 경우 연구를 중단하고 필요한 조치를 취하게 될 것입니다.

연구 참여 여부와 관계없이, 시술을 위한 전신마취 중 인공호흡기를 통한 산소요법은 반드시 필요한 것으로, 대안이 있는 것이 아니며, 마취 시 필요에 따라 흡입산소분율을 조정하게 됩니다. 본 연구는 이 흡입산소분율이 심근 손상 지표에 영향을 미치는지 살펴보기 위한 것입니다. 본 연구에서 동맥산소분압 및 심근 손상의 지표를 측정하기 위해 시행되는 검사는 본 연구와 상관 없이 통상적인 대동맥판막 치환술 환

자에서 시행되는 검사의 종류 및 범위와 크게 다르지 않으며, 본 연구를 위해 추가적으로 시행되는 검사 비용에 대해서는 대상자분이 부담하지 않을 것입니다.

7. 연구대상자에게 예견되는 이득

임상연구를 통하여 대상자에게 기대되는 직접적인 이득은 없으나, 연구를 통해 경피적 대동맥판막 치환술을 위한 마취 중 적절한 산소화에 대한 지표가 확립될 수 있도록 이론적 근거가 마련된다면 향후 안전한 마취 제공에 도움이 될 수 있을 것으로 기대할 수 있습니다.

8. 연구 참여 비용 및 손실에 대한 보상

연구에 참여함으로써 대상자에게 추가로 드는 비용은 없으며, 임상연구와 관련하여 손실이 발생하였을 경우 인과관계에 따라 적절한 보상이 주어질 것입니다.

9. 자발적 참여 및 동의 철회

본 연구에의 참여 여부 결정은 자발적 참여를 원칙으로 하고 있으며, 연구에의 참여 동의 후에라도 대상자가 연구 참여를 거부하거나 동의를 철회하고자 할 때에는 언제라도 연구에서 제외될 수 있으며, 연구에 참여하지 않는다고 하여 환자분이 원래 받을 수 있는 이익에 대한 손실은 발생하지 않을 것입니다. 연구에 참여하기로 하였더라도 시술 계획의 일부 변경 등으로 연구 대상자의 선정 기준에 부합하지 않거나, 이전에 알려지지 않았던 심각한 기저 질환의 발견 등으로 연구 제외 기준에 들게 되는 경우, 또는 시술 중 연구에 포함된 검사나 처치를 지속하기 어려운 점이 발견될 때에는 연구 도중 연구에의 참여가 중지될 수 있습니다. 또한, 이외에도 대상자의 임상시험 참여 지속 여부 결정 변경에 영향을 줄 수 있는 새로운 정보를 취득할 경우, 즉시 대상자 또는 대상자의 대리인에게 고지할 것입니다.

10. 개인정보보호 및 개인정보 제공에 관한 사항

본 연구에 참여하는 대상자의 신상을 파악할 수 있는 모든 기록은 비밀로 보호될 것이며, 연구의 결과가 출판될 경우 대상자의 신상은 비밀로 보호될 것입니다. 본 연구 결과는 학술 목적으로만 이용되며 학술적인 보고를 위해 외부로 발표될 수 있습니다. 대상자의 성별, 나이, 체중, 신장 등의 의무기록 정보가 연구에 사용되나 대상자와 담당 의사, 그리고 연구에의 참여자 이외에는 대상자의 연구 참여 여부나 치료 경과에 대해서는 알지 못할 것입니다. 환자분의 검진 기록은 비밀이 유지되고 다른 곳으로 이동되지 않을 것이며, 본 연구의 진행 여부를 감독 받기 위해 감독 기관으로 보내어 질 수 있습니다. 임상시험 진행 중 및 연구시험 종료 후에도 본 연구의 모니터 요원, 점검을 실시하는 사람, 의학연구윤리심의위원회 및 보건복지부장관 등이 관계 법령에 따라 연구의 실시 절차와 자료의 품질을 검증하기 위해 대상자의 신상에 관한 비밀이 보호되는 범위에서 대상자의 연구 기록을 열람할 수 있으며 대상자 또는 대상자의 대리인이 서명한 동의서에 의하여 이러한 자료의 열람이 허용될 수 있습니다. 모든 기록은 법이 정하는 기간 동안 보관되며 추후 모든 자료는 안전하게 폐기됩니다

11. 담당자 연락처

임상연구에서 발생한 문제, 우려, 연구대상자의 권익에 대한 문제, 우려, 질문에 대하여 아래 담당자 또는 서울대학교병원 의학연구윤리심의위원회(전화 02-2072-0694, 전자우편 snuhirb@gmail.com) 또는 임상연구윤리센터(전화 02-2072-3509)로 연락하여 주시기 바랍니다.
본 설명문과 동의서의 사본이 대상자분께 제공될 것입니다.

연구 책임자: 서울대학교병원 마취통증의학과 교수 전윤석 02-2072-2465
연구 담당자: 서울대학교병원 마취통증의학과 진료교수 조연정 02-2072-3108

대상자 일련번호	

연구대상자 동의서

경피적 대동맥판막 치환술 중 산소화 정도가 심근 손상에 미치는 영향

1. 본인은 임상연구에 대해 구두로 설명을 받고 상기 연구 설명문을 읽었으며 담당 연구원과 이 연구에 대하여 충분히 의논하였습니다.
2. 본인은 연구의 위험과 이득에 관하여 들었으며 나의 질문에 만족할 만한 답변을 얻었습니다.
3. 본인은 이 연구에 참여하는 것에 대하여 자발적으로 동의합니다.
4. 본인은 이후의 치료에 영향을 받지 않고 언제든지 연구의 참여를 거부하거나 연구의 참여를 중도에 철회할 수 있고 이러한 결정이 나에게 어떠한 해가 되지 않을 것이라는 것을 알고 있습니다.
5. 본인은 이 설명서 및 동의서에 서명함으로써 의학 연구 목적으로 나의 개인정보가 현행 법률과 규정이 허용하는 범위 내에서 연구자가 수집하고 처리하는데 동의합니다.
6. 본인은 연구 설명문 및 동의서의 사본을 받을 것을 알고 있습니다.

연구대상자 성명	서명	날짜(년/월/일)
시험자/연구자 성명	서명	날짜(년/월/일)
법정대리인 성명	서명	날짜(년/월/일)
(대상자와 대리인과의 관계)	(대리인 동의 사유)	

[별첨2] 증례기록서

경피적 대동맥판막 치환술 중 산소화 정도가 심근 손상에 미치는 영향

증례번호: ________________ 날짜: ______________________________ 동의서 여부 ( )

Patient Information											
Sex	☐ M ☐ F	Age		Ht		Wt		BMI		BSA	

Pre-procedure Dx		emergency	☐ Y ☐ N
Post-procedure Dx		pregnancy	☐ Y ☐ N
Procedure name			

Patient Past Medical History			
☐ HTN		☐ Poorly controlled HTN? ☐ Y ☐ N	
☐ DM		☐ Poorly controlled DM? ☐ Y ☐ N	
☐ Renal dysfunction ☐ Mild-Moderate ☐ Severe		☐ Dialysis ☐ Hemodialysis ☐ Peritoneal dialysis	
☐ Chronic liver disease ☐ Mild-Moderate ☐ Severe		☐ Tb ☐ Asthma ☐ COPD	
☐ Baseline PaO_2	mmHg	☐ Mechanically ventilated? ☐ Y ☐ N	
☐ Baseline serum creatinine	mg / dL		
☐ Baseline MDRD GFR	mL/min/1.73m^2		
☐ Smoking: ☐ never ☐ current ☐ former			
☐ Previous ACS(6 개월 이내) ☐ Y ☐ N		☐ Recent PCI (1 개월 이내)? ☐ Y ☐ N	
☐ Stroke or TIA within 6 mo? ☐ Y ☐ N		☐ Coronary artery disease? ☐ Y ☐ N	
ASA class		STS score (mortality risk)	%

Preop PFT	FEV_1 (%)	FVC (%)	FEV_1 / FVC %

Current Medication			
BB	☐ Y ( ) ☐ N	aspirin	☐ Y ☐ N
ARB	☐ Y ( ) ☐ N	anti-platelet agents	☐ Y ☐ N
ACEi	☐ Y ( ) ☐ N	종류/ 용량	
CCB	☐ Y ( ) ☐ N		
Diuretics	☐ Y ( ) ☐ N	iv heparin	☐ Y ( ) ☐ N
Oral Nitrates	☐ Y ( ) ☐ N	iv NTG	☐ Y ( ) ☐ N
DGX	☐ Y ( ) ☐ N	LMWH	☐ Y ( ) ☐ N
Statin	☐ Y ( ) ☐ N	Insulin	☐ Y ( ) ☐ N
OHA	☐ Y ( ) ☐ N		
Preoperative Inotropics or other drugs			

Hemodynamics							
	마취유도 전	마취유도 10분 후	Valve implatation 후	End of procedure	FiO2/~~flow~~ 조정 10 분 후 (1)	FiO2/~~flow~~ 조정 10 분 후 (2)	FiO2/~~flow~~ 조정 10 분 후 (3)
FiO2/~~flow~~ 조정 사항							
FiO2/~~flow~~ 조정 시점							

HR (min)							
SBP (mmHg)							
DBP (mmHg)							
MBP (mmHg)							
CVP (mmHg)							
SPAP (mmHg)							
DPAP (mmHg)							
MPAP (mmHg)							
SpO_2 (%)							
BT (℃)							
SVV (%)							
SvO_2 (%)							
Cardiac index (L/m^2/min)							
Cardiac output (L/min)							
Cerebral oximeter (%, Lt/ Rt)							
BIS							
PPF							
remiFTN							

Laboratory Data (1)								
		**마취유도 전**	**마취유도 10분 후**	**Valve implatation 후**	**End of procedure**	**FiO2/~~flow~~ 조정 10 분 후 (1)**	**FiO2/~~flow~~ 조정 10 분 후 (2)**	**FiO2/~~flow~~ 조정 10 분 후 (3)**
**FiO2 조정 사항**								
**FiO2 조정 시점**								
ABGA	Hct (%)							
	pH							
	PaO2 (mmHg)							
	PaCO2 (mmHg)							
	SaO2 (%)							
	BE (mmol/L)							
	BEecf (mmol/L)							
	Lactic acid (mmol/L)							

Laboratory Data (2)							
	**Baseline (시술전)**	**post-procedure 0h**	**post-procedure 4h**	**post-procedure 8h**	**pod1**	**pod2**	**pod3**
CK-MB (ng/mL)							
Tn I (ng/mlL)							
Cr (mg/dL)							
Hb (g/dL)							
Hct (%)							
Albumin (g/dL)							
ALP (IU/L)							

hs-CRP (mg/dL)							

Procedural Information							
시술시간	분	마취시간	분	총 PPF	mg	총 remiFTN	μg
crystalloid	mL	colloid	mL	U/O	mL	EBL	mL
**Baseline Echocardiographic findings**							
LVEF	%	LVIDd	mm	LVIDs	mm	LV mass	g
AVA	cm^2	AV peak vel	m/s	AV mean PG	mmHg	AS degree	
PASP	mmHg						
Intra-procedure event							
Valve 종류		Size	mm	PVL degree		Block	
Inotropic requirements (시술 후 72 시간까지)							
**Transfusion**							
Pre-procedure (prev 7d) ☐ RBC (_____ u) ☐ FFP (_____ u) ☐ PLT (_____ u) ☐ Cryo (_____ u)							
Intra-procedure ☐ RBC (____ u) ☐ FFP (____ u) ☐ PLT (____ u) ☐ Cryo (____ u) ☐ Cell saver (_____ mL)							
Post-procedure (72 hr) ☐ RBC (_____ u) ☐ FFP (_____ u) ☐ PLT (_____ u) ☐ Cryo (_____ u)							
**Peri-procedure MACCEs (Major Cardiovascular & Cerebral Events)**							
☐ Death				☐ Peri-procedure myocardiac infarction			
☐ Newly developed stroke / TIA				☐ New continuous renal replacement therapy			
☐ Newly developed AKI				☐ Newly developed cardiac arrhythmia / block			
Mechanical Ventilation time	분	CCU stay	hr	Post-procedure hospital stay	일		
Other coplications							
**Post-procedural delirium**							
CAM-ICU	☐ 양성 ☐ 음성	양성일 경우, 시술 후 일수 : POD ( ), POD ( ), POD ( ), POD ( )					
최종 양성의 판정 : 특성 1 과 2 가 양성이고, 특성 3 또는 4 중 1 개 이상 양성인 경우							
특성 1	급성으로 발생하였거나 계속 변화하는 경과인가?						
특성 2	주의력 결핍						
특성 3	의식 수준의 변화						
특성 4	비체계적인 사고						

Post-procedure O2 therapy			
**Type**		**POD1 PaO2 / SaO2**	
**Time**		**POD2 PaO2 / SaO2**	
**FiO2**		**POD3 PaO2 / SaO2**	

기타 특이사항

이상 반응 평가

이상 반응	중대성	중증도	기간	인과관계

**임상 시험 책임자 서명**					
본인은 위의 대상자로부터 적절한 방법에 따라 문서 동의를 얻었으며, 본 증례기록지에 기록된 모든 내용이 위의 대상자로부터 정확히 얻어진 결과임을 확인합니다.<br>본인 또는 본인이 위임하는 자는,<br>1. 본 증례 기록지의 내용을 모두 검토하였으며,<br>2. 그 내용이 정확하고<br>3. 기록된 날짜에 시행된 검사 또는 측정의 결과임을 확인합니다.					
시험자 성명		서명		날짜	년 월 일

대상자 등록일: ________________년 _________월 _________일
동의일: ________________년 _________월 _________일

[별첨3] 임상시험 피해자 보상규약

본 연구는 경피적 대동맥판막 치환술 중 산소화 정도에 따른 시술 후 심근 손상의 지표의 차이를 알아보고자 하는 연구로서, 기존 진료에서 진행되고 있는 시술 및 치료 방법 등에 대한 연구입니다. 의학적으로 판단하여, 연구로 인한 추가적 위험이 기존 진료 과정에서 진행되고 있는 시술, 또는 치료 방법 보다 현저하지 않으므로, 이 연구로 인해 연구대상자가 추가적으로 입게 되는 신체적, 정신적 위해 및 특이 손상은 없을 것으로 예측됩니다.
연구대상자들에게는 통상적인 진료 과정에서 이루어지는 안전 보호 대책이 적용될 것이며, 연구 시작 전 연구대상자들에게 해당 연구의 목적과 방법 등에 대한 충분한 정보가 제공될 것입니다. 또한, 임상시험 도중, 기존 진료 과정 외 임상시험 참여로 추가된 절차 및 개입으로 인해, 예측한 또는 예측되지 못한 이상반응 등이 발생할 경우, 연구대상자가 적절한 의학적 처치를 받을 수 있도록 가능한 최선의 치료를 시행 할 것이며, 임상시험과 손상 사이의 합리적인 인과관계가 있는 경우 연구대상자에게 보상하겠습니다.
연구 책임자는 본 연구 시험 실시 중 본 연구의 직접적인 원인에 의해서 초래된 합병증에 대하여 치료가 필요한 경우 연구대상자의 부담이 최소화되도록 노력할 것입니다.

1. 합병증의 처리는 다음 사항에 적합하여야 합니다.
1) 연구대상자는 본 시험의 계획서를 충실히 이행하여야 합니다.
2) 발생한 합병증에 대해서는 연구 책임자, 담당자, 공동 연구자에 즉시 연락을 취하여야 합니다.

2. 다음 경우에는 보상에서 제외됩니다.
1) 연구 책임자, 담당자, 공동 연구자의 후원 하에 집행되지 않았거나 제고하지 않은 의약품 등으로 발생한 이상반응에 의한 손상
2) 임상 연구에 의한 효과 또는 혜택을 제공하지 못한 데 대한 보상
3) 서로 합의한 연구실험계획서를 시행하지 않음으로 야기된 손상
4) 연구대상자의 부주의에서 초래된 손상
5) 질병의 자연 경과에 의해 발생한 손상
6) 본 연구가 아니더라도 연구대상자에게 시행되었어야 할 수술 등 치료에 의해 발생한 손상

3. 피해 구제 기준
상기한 바에 의하여 연구대상자에 치료가 필요한 경우 환자의 부담이 최소화하도록 노력할 것입니다.

앞에서 언급한 여러 제반 내용을 참고하여 연구대상자가 본 시험에 의해 어떤 불이익도 받지 않도록 할 것이며 연구대상자가 연구 도중 언제라도 중도에 이익에 대한 손실 없이 참여를 포기할 수 있고 연구대상자가 본 시험에 참여하지 않아도 어떠한 불이익도 받지 않도록 주의할 것을 서약합니다.

날짜: 2017년 5월 16일
책임연구자: 전윤석 (서명)

[별첨4] 책임연구자의 이력

성명: 전윤석
2020년 12월 19일 임상연구윤리(GCP)교육을 이수함.

학력

학위과정명	기 간		학부(과)	전공	학위명	대학(원) 수여기관
	입학연월일	졸업연월일				
학 사	1988.3	1994.2	의학과	의학과	의학사	서울대학교
석 사	1998.3	2003.2	의학과	마취과학	의학석사	서울대학교
박 사	2003.3	2005.2	의학과	마취과학	의학박사	서울대학교

※ 의사면허번호 : 53042 (취득일 1994.3), 전문의자격번호: 2013 (취득일 1999.3)

경력

기 간	근 무 기 관 명	직 위	담 당 업 무
2017.3~	서울대병원	교수	임상진료
2012.3~2017.2	서울대병원	부교수	임상진료
2008.3~2012.2	서울대병원	기금조교수	임상진료
2005.5~2008.2	서울대병원	임상교수	임상진료
2004.5～2005.4	서울대병원	촉탁의	임상진료
2002.5～2003.4	서울대병원	전임의	임상진료
1999.5～2002.4	서귀포성심병원	과장	임상진료
1995.3～1999.2	서울대병원	전공의	
1994.3～1995.2	서울대병원	인턴	

학회 발표 논문

제 목	발 표 년월일	발 표 학회/장소	분류	저자	
				인원	공동연구 내역
Localization of the catheter tip via the right artial electrocardiography: Transeophaeal Echocardiographic Confirmation	2003/10	American Society of Anesthesiologists Annul Meeting/ San Francisco	논문	5	1저자

A novel method to maintain high FiO2 while using self inflating resuscitator with high minute ventilation.	2004/5/17	Society of Cardiovascular Anesthesiologist Annual Meeting/ Hawaii	논문	8	1저자
Comparative hemodynamic effects of vasopressin and norepinephrine after milrinone-induced hypotension in off-pump coronary artery bypass surgical patients	2004/9/9-12	9th Internaltional Congress of Cardiothoracic and Vascular Anesthesia/ Tokyo	논문	7	책임저자
Does Modified Thromboelastogram Could Assess Platelet Inhibition by Aspirin in Healthy Volunteer and OPCAB patient?	2004/11/6	대한마취과학회 제49차 학술대회/서울	논문	6	책임저자
Collagen Whole Blood Aggregometry Predicts Myocardial Injury after off-Pump Coroanry Bypasss Grafting in Aspirin Taking Patients	2005/11/18	대한마취과학회 제50차 학술대회/제주	논문	3	책임저자
대동맥파열수술이후 혈액투석을 받은 환자에서 발생한 헤파린기인성 혈소판감소증	2005/11/18	대한마취과학회 제50차 학술대회/제주	논문	6	책임저자
Experiences of Off-pump Coronary Artery Revascularization in Awake Patients	2005/11/18	대한마취과학회 제50차 학술대회/제주	논문	7	책임저자
Anesthetic Consideration in ACAB	2005/12/10	2005 Update in Coronary Artery Surgery/세종병원	Invited Lecture		
The Effect of Morphine Preconditioning on Brain Focal Ischemia in Rat by Middle Cerebral Artery Occlusion	2005/5/17	Society of Cardiovascular Anesthesiologists Annual Meeting / Baltimore	논문	3	제1저자
Transesophagel Echocardiography in Operating Room	2005/9/11	Echo Seoul 2006/서울	Invited Lecture		
체외순환을 이용하지 않는 관상동맥우회술 중 발생한 중증의 승모판막 전판막의 수축기 전이동	2006/4/8	대한심폐마취학회/대구	증례	6	책임저자

체외순환을 하지 않는 관상동맥우회술 중 고유량의 이산화탄소 통기에 의해 발생한 대량의 이산화탄소 정맥 색전증	2006/4/8	대한심폐마취학회/대구	증례	6	책임저자
The effect of flexion and extension of cervical spine on the distribution of contrast medium at high thoracic epidural space	2006/10	American Society of Anesthesiologists Annul Meeting/ Chicago	논문		책임저자
Intravenous palonosetron increases prolonged QT patients during sevoflurane general anesthesia for laparotomy	2012/10	American Society of Anesthesiologists Annul Meeting/ Washington	논문	5	책임저자
Effects of palonosetron on perioperative cardiovascular complications in patients undergoing noncardiac surgery with general anesthesia: A retrospective cohort study.	2013/03	Society of Cardiovascular Anesthesiologists Annual Meeting / Miami	논문	5	책임저자
Association between red blood cell storage duration and clinical outcome in patients undergoing off-pump coronary artery bypass surgery: a retrospective study	2014/10	American Society of Anesthesiologists Annul Meeting/New Orleans	논문	8	책임저자

학회지 발표 논문

제 목	발 표 년월일	발 표 지	분류	저자	
				인원	공동연구 내역
개심술 환자에서 술후 기관내 삼관 기간에 관한 고찰	1997.10	대한마취과학회지	논문	6	공저자
통원 수술 환자의 되원 기준으로서 PAR Score와 Modified PADSS의 유용성	1998.11	대한마취과학회지	논문	7	1저자
심실부수축기를 가진 환자의 마취 중 발견된 간헐적 조기흥분 증후근	1999.12	대한마취과학회지	증례	5	1저자
경막외강 스테로이드 주입술 시행 시 주입된 공기에 의해 발생한 뇌간 압박	2000.8	대한마취화학회지	증례	2	1저자
허혈성 심근병증 환자에서 Endoventricular Circular Patch Plasty 수술 중 및 술 후 환자관리	2002	대한중환자의학회지	논문	8	공저자
Intravenous clonidine prolongs bupivacaine spinal anesthesia	2003.9	Acta Anaesthesiol Scan	SCI	4	공저자

Prophylactic milrinone during OPCAB of posterior vessels: implication in angina patients taking beta-blockers	2003.11	Europeran Journal of Cardio-thoracic Surgery	SCI	7	공저자
수술전 심장초음파 소견과 심폐우회술 없이 시행하는 관상동맥 우회술 후 예후 판정에 대한 유효성	2003.4	대한마취과학회지	학위 논문	5	제1저자
관상동맥 우회술을 받는 환자에서 Milrinone과 저용량 Vasopressin 정주에 의한 혈역학적 효과	2004.3	대한마취학회지	논문	7	제1저자
Selection of an endotracheal tube for device-guided intubation	2003.5	Anesth Analg	letter	2	
Could Modified Thromboelastogram Assess Platelet Inhbition by Aspirin in off Pump Coronary Artery Bypass Surgical Patients and Healthy Volunteers?	2004.12	대한마취과학회지	논문	6	책임저자
A New Technique to Determine the Size of Double-lumen Endotracheal Tubes by the Two Perpendicularly Measured Bronchial Diameters	2005.2	Anaesth Intensive Care	SCI	5	제1저자
Intrathecal clonidine does not reduce post-spinal shivering	2005.3	Acta Anesthesiol Scan	SCI	6	1저자
흉부 대동맥 질환에서 내혈관적 스텐트 이식편 삽입시의 마취경험(증례)	2005.7	대한마취과학회지	증례	4	공저자
고용량의 환기량에서 인공호흡낭을 이용해 흡입 산소 농도를 유지하는 방법	2005.3	대한마취과학회지	논문	6	책임저자
심폐체외순환을 포함한 심장수술 이후 혈액투석을 받은 환자에서 발생한 Heparin 기인성 혈소판 감소증	2006.5	대한마취과학회지	증례	10	책임저자
Comparative hemodynamic effects of vasopressin and norepinephrine after milrinone-induced hypotension in off-pump coronary artery bypass surgical patients	2006.6	European Journal of Cardio-thoracic Surgery	SCI	7	제1저자
경막외 마취를 이용한 각성하 관상동맥우회술	Accepted	대한마취과학회지	논문		책임저자
Transesophageal Echocardiographic Evaluation of the Electrocardiography-Guided Localization of Central Venous Catheter	2006.10	Canadian Journal of Anestheisa	SCI	5	제1저자

The carina as a useful radiographic landmark for positioning the intraaortic balloon pump	2007.9	Anesthesia and Analgesia	SCI	11	공저자
Massive carbon dioxide embolism caused by a carbon dioxide blower during the repair of a coronary vein during off-pump coronary artery bypass.	2007.10	Journal of Cardiothoracic and Vascular Anesthesia	증례	5	책임저자
Ultrasonographic investigation of the effect of inguinal compression on the cross-sectional area of the femoral vein	2008.1	Academic Emergency Medicine	SCI	7	공저자
Comparison of thoracic epidural pressure in the sitting and lateral decubitus positions	2008.7	Anesthesiology	SCI	6	공저자
The head-down tilt position decreases vasopressor requirement during hypotension following induction of anaesthesia in patients undergoing elective coronary artery bypass graft and valvular heart surgeries	2011.1	opean Journal of Anaesthesiology	SCI	9	공저자
Pulse pressure variation as a predictor of fluid responsiveness during one-lung ventilation for lung surgery using thoracotomy: randomised controlled study	2011.1	European Journal of Anaesthesiology	SCI	7	책임저자
Pulse-pressure variation predicts fluid responsiveness during heart displacement for off-pump coronary artery bypass surgery	2011.12	Journal of Cardiothoracic and Vascular Anesthesia	SCI	7	책임저자
Effect of the bevel direction of puncture needle on success rate and complications during internal jugular vein catheterization.	2012.02	Critical Care Medicine	SCI	5	공저자
Effects of remote ischemic preconditioning with postconditioning in patients undergoing off-pump coronary artery bypass surgery--randomized controlled trial	2012	Circulation Journal	SCI	8	책임저자
Does remote ischaemic preconditioning with postconditioning improve clinical outcomes of patients undergoing cardiac surgery? Remote Ischaemic Preconditioning with Postconditioning Outcome Trial.	2014.1	European Heart Journal	SCI	10	책임저자

Anesthetic management of antiphospholipid syndrome patients who underwent cardiac surgery: three cases report.	2014.2	Korean Journal of Anesthesiology	증례	5	책임저자
A randomised controlled trial comparing incentive spirometry with the Acapella® device for physiotherapy after thoracoscopic lung resection surgery	2014.8	Anaesthesia	SCI	11	책임저자
Evaluation of a simplified augmented reality device for ultrasound-guided vascular access in a vascular phantom	2014.9	Journal of Clinical Anesthesia	SCI	3	책임저자
Association between red blood cell storage duration and clinical outcome in patients undergoing off-pump coronary artery bypass surgery: a retrospective study	2014.10	BMC Anesthesilogy	SCI	8	책임저자
Increased tracheal cuff pressure during insertion of a transoesophageal echocardiography probe: A prospective, observational study	2015.1	European Journal of Anaesthesiology	SCI	8	책임저자
Tissue microcirculation measured by vascular occlusion test during anesthesia induction	2015.03	Journal of Clinical Monitoring and [illegible]	SCI	7	책임저자
Effects of palonosetron on perioperative cardiovascular complications in patients undergoing noncardiac surgery with general anesthesia: A retrospective cohort study.	2015.03	Clinical Pharmacology and Therapeutics	SCI	10	책임저자
Microvascular reactivity and clinical outcomes in cardiac surgery	2015.09	Critical Care	SCI	7	책임저자
Comparison of Needle Insertion and Guidewire Placement Techniques During Internal Jugular Vein Catheterization: The Thin-Wall Introducer Needle Technique Versus the Cannula-Over-Needle Technique	2015.10	Critical Care Medicine	SCI	9	공저자
Comparison of cardiac output measures by transpulmonary thermodilution, pulse contour analysis, and pulmonary artery thermodilution during off-pump coronary artery bypass surgery: a subgroup analysis of the cardiovascular anaesthesia registry at a single tertiary centre	2015.10	Journal of Clinical Monitoring and Computing	SCI	5	책임저자

Misplacement of left-sided double-lumen tubes into the right mainstem bronchus: incidence, risk factors and blind repositioning techniques	2015.10	BMC Anesthesiology	SCI	6	공저자
Pulmonary Protective Effects of Remote Ischaemic Preconditioning with Postconditioning in Patients undergoing Cardiac Surgery involving Cardiopulmonary Bypass: A substudy of the Remote Ischaemic Preconditioning with Postconditioning Outcome trial	2015.10	Heart, Lung & Circulation	SCI	11	책임저자
Intraoperative anaphylaxis to neuromuscular blocking agents: the incidence over 9 years at two tertiary hospitals in South Korea: A retrospective observational study	2015.11	European Journal of Anaesthesiology	SCI	11	책임저자
Efficacy of intrathecal morphine for postoperative pain management following open nephrectomy	2016.2	The journal of international medical research	SCI	7	공저자
